# Contact calls in woodpeckers are individually distinctive, show significant sex differences and enable mate recognition

**DOI:** 10.1038/s41598-021-02034-3

**Published:** 2021-11-23

**Authors:** Ewa Węgrzyn, Wiktor Węgrzyn, Konrad Leniowski

**Affiliations:** 1 Institute of Biology & Biotechnology, University of Rzeszów, Rejtana 16C, 35-959 Rzeszów, Poland; 2 Faculty of Health Sciences, Medical University of Lodz, Pl. gen. J. Hallera 1, 90-647 Łódź, Poland

**Keywords:** Ecology, Evolution, Zoology

## Abstract

Vocal communication of woodpeckers has been relatively little studied so far, mostly because majority of species use drumming to communicate. Our recent study on the Middle Spotted Woodpecker revealed that a call which is specific for floaters is individually distinctive and functions as a vocal signature of unpaired individuals. The aim of the current study is to investigate whether a contact call of paired territory owners of the same species enables discrimination of individuals and their sex. Acoustic analyses revealed that the call is individually distinctive and experimental approach confirmed that woodpeckers are able to distinguish between a contact call of their partner and a stranger. We also found that the contact call shows significant sex differences. Interestingly, the acoustic parameter enabling sex identification is different than the parameters coding individual variability of the call. The design of a call so that its first part would code the identity of an individual and the second part would code its sex presents an effective and fine-tuned communication system. The results of our study also suggest that the contact call of paired Middle Spotted Woodpeckers may be useful for conservation biologists as a tool supporting other census methods.

## Introduction

Vocal communication plays an important role across bird species. It helps to resolve territory conflicts and defend boundaries against rival males as well as to attract females^1–3^. There is a vast evidence of between-individual variability in vocal traits of individuals belonging to the same species or even population^4–7^. This variability exists in many aspects of bird vocal behavior and may have evolved for different aims, such as quality advertisement^8–10^ or individual recognition^11,12^. In the latter case vocal distinctiveness results from slight differences in a syrinx and airsacks structure or the length and movements of a beak^13–15^. A certain combination of physical traits often produces vocal signatures of individuals. Numerous social interactions depend on proper recognition of individuals, for example mates^16–20^, neighbours^6,21–26^ or offspring^16,27–29^. It also helps to maintain dominance hierarchies^30,31^ and to avoid inbreeding^32^. Thus, one may infer that vocal signatures and the recognition of individuals using spectral characteristics of their voice should be widely prevalent in avian taxa due to numerous benefits both for signalers and receivers.

As it comes to woodpeckers, their vocal communication has been relatively little studied so far, mostly because majority of woodpecker species use drumming in mate attraction and territory defence^33–35^. Having said that, calls may play an important role in woodpeckers communication^36,37^ and some species, like for example the Middle Spotted Woodpecker *Dendrocoptes medius*, drum rarely and use mainly calls to communicate.

Our recent study, based on observation of spontaneous interactions between individuals and playback experiments, revealed that the Middle Spotted Woodpecker produces three call types of different spectral characteristics and functions^12^. Call-1 and Call-2 are characteristic for paired individuals, whereas Call-3 is typical for floaters. Call-1 is generally produced by individuals sitting in a tree. Territorial pairs use Call-1 as a contact call and an initial response to any disturbance within a territory. Call-2 is frequently uttered by territory owners during flight toward intruder and it goes along with territorial conflicts and trials to remove a rival from a territory. It frequently precedes a direct fight. Call-3, typical for unpaired individuals, is a persistent vocalization without any obvious stimulus. Spectral characteristics of call-3 proved to be individually distinctive and play-back experiments revealed that woodpeckers were able to distinguish between calling floaters—calls of familiar floaters induced a reduced aggression of territory owners compared to calls of stranger floaters. This led to the conclusion that Call-3 functions as a vocal signature of unpaired individuals.

Our current study aims to test whether calls of paired Middle Spotted Woodpeckers are also individually distinctive and enable recognition of particular conspecifics. We have analyzed bioacoustics parameters of Call-1 produced by different territorial individuals to find out whether this call may function as a vocal signature of paired individuals. We have also investigated the reaction of pair-members to a play-back of Call-1 uttered by their partner and by a stranger. Call-1 is often used as a contact call between pair members when they forage separately in different parts of a territory^12^, thus it seems logical that mates should be able to distinguish calls of their partner from the vocalization of neighboring Middle Spotted Woodpecker pairs or intruders. We test the hypotheses that (i) between-individual differences in spectral characteristics of Call-1 are greater than within-individual variation in the production of this vocalization, (ii) individuals respond differently to Call-1 produced by their mate and unfamiliar Middle Spotted Woodpecker and (iii) spectral characteristics of Call-1 show significant sex differences and enable to tell males from females. The latter assumption, if confirmed, would be a valuable tool for researchers studying this species because sexual dimorphism is hardly visible in the Middle Spotted Woodpecker and males can be distinguished from females only by experienced field workers by slight differences in the length of the red crown^38^. We also inquire which structures of the contact call of woodpeckers convey information about identity and sex of calling individuals. We hypothesize that even a quite simple waveform pattern used by the Middle Spotted Woodpecker as a contact call is sufficient to encode a multidimensional message.

## Results

**Figure 1:**
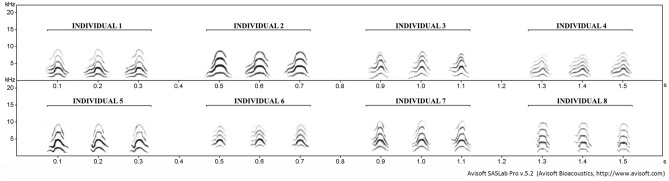
Between-individual differences of Call-1 produced by 8 different individuals. Individuals 1–4 (in upper row) are males and individuals 5–8 (in bottom row) are females. To show the consistency of Call-1 produced by the same individual three examples of Call-1 extracted from the same bird are presented for each individual.

Visual analysis of sonograms of Call-1 of different individuals initially supported our hypothesis that between-individual differences in spectral characteristics of Call-1 are greater than within-individual variation in the production of this vocalization (Fig. 1).

Subsequent spectral analyses of Call-1 produced by 31 different individuals confirmed the outcomes of visual inspection of sonograms—5 out of 8 analyzed parameters of Call-1 (described in Methods section) differed significantly between individuals (Table 1), which indicates that Call-1 is individually distinctive.

**Table 1:** ANOVA test for the between-individual differences in eight parameters of Call-1, *n* = 31 individuals, 310 calls.

Acoustic parameters	$\overline{x}$ ± SD	F	*p*
duration of a Call-1 [s]	0.047 ± 0.009	8.206	** < 0.001**
(A) frequency of the beginning of the dominant frequency [Hz]	2021 ± 601	1.921	** < 0.001**
(B) frequency of the mid-part of the dominant frequency [Hz]	4079 ± 393	1.904	**0.002**
(C) frequency of the end of the dominant frequency [Hz]	2009 ± 486	1.319	0.093
(D) frequency of the mid-part of the fundamental frequency [Hz]	2119 ± 486	2.104	**0.001**
(A/B) the change in the frequency of the first part of the dominant frequency	0.50 ± 0.14	2.296	** < 0.001**
(C/B) the change in the frequency of the second part of the dominant frequency	2.14 ± 0.55	1.224	0.101
(A/B–C/B) the change in the frequency of the whole dominant frequency	− 0.002 ± 0.15	0.982	0.518

**Figure 2:**
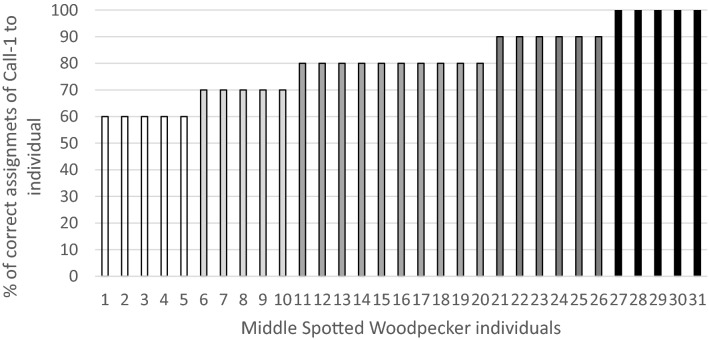
The level of correct classifications of Call-1 to individuals (*n* = 31 individuals, 310 calls). Individuals were ordered according to growing level of correct classifications.

Discriminant analysis (DFA) showed high accuracy of proper classification of 310 calls to 31 individuals based on the selected eight acoustic parameters of Call-1. Cross-validated procedure resulted in the correct assignment of calls to individual woodpeckers in 80.3% of all cases. This value is well above random assignment (3.2%), which shows that Call-1 is individually distinctive and may function as a vocal signature of paired woodpeckers. The lowest score of correct classification of calls to an individual was 60%, and the highest score was 100% (Fig. 2). The most frequent score of correct classifications was 80%.

**Figure 3:**
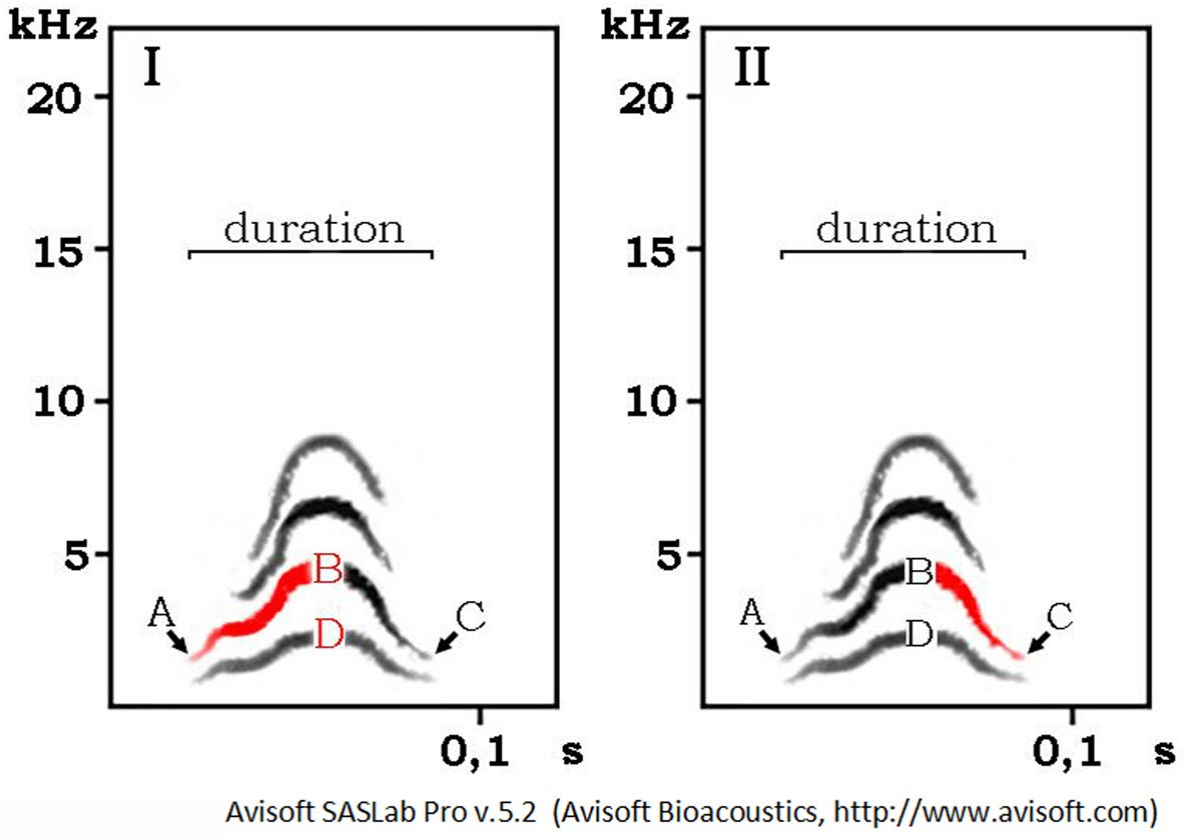
Sonograms of Call-1 with acoustic parameters (marked in red) that explained the highest level of: I. between individual variability (B—frequency of the mid-part of the dominant frequency, A/B—the change in the frequency of the first part of the dominant frequency, D—frequency of the mid-part of the fundamental frequency) II. between-sex variability (C/B—the change in the frequency of the second part of the dominant frequency).

Post-hoc analysis revealed that the acoustic parameters that explained the highest level of between-individual variability in Call-1 were: frequency of the mid-part of the fundamental frequency (63.6%), the change in the frequency of the first part of the dominant frequency (13.8%) and frequency of the mid-part of the dominant frequency (12.5%), which together explained 90% of differences (Fig. 3A).

We also compared the selected eight acoustic parameters between sexes. We found significant differences between males and females in six out of eight analyzed parameters of Call-1 (Table 2).

**Table 2:** T-test for between-sex differences in eight parameters of Call-1, *n* = 24 individuals (10 females and 14 males), 240 calls.

Acoustic parameters	$\overline{x}$ ± SD	t	*p*
duration of a Call-1 [s]	♀0,44 ± 0,007	− 3,43	**0,001**
	♂0,48 ± 0,010		
(A) frequency of the beginning of dominant frequency [Hz]	♀1995 ± 325	− 0,54	0,589
	♂2038 ± 796		
(B) frequency of the mid-part of the dominant frequency [Hz]	♀3948 ± 372	− 3,27	**0,001**
	♂4109 ± 378		
(C) frequency of the end of the dominant frequency [Hz]	♀2132 ± 477	4,18	** < 0,001**
	♂1872 ± 471		
(D) frequency of the mid-part of the fundamental frequency [Hz]	♀2049 ± 214	− 2,12	**0,035**
	♂2120 ± 283		
(A/B) the change in the frequency of the first part of the dominant frequency	♀0,51 ± 0,10	0,87	0,387
	♂0,50 ± 0,17		
(C/B) the change in the frequency of the second part of the dominant frequency	♀1,90 ± 0,34	− 5,92	** < 0,001**
	♂2,34 ± 0,67		
(A/B–C/B) the change in the frequency of the whole dominant frequency	♀0,03 ± 0,12	3,28	**0,001**
	♂-0,04 ± 0,17		

Subsequent discriminant analysis (DFA) with cross-validated procedure demonstrated a correct assignment of calls to sex in 79.2%. Proper classification of female calls was 80% and male calls 78.6%. Obtained values show that Call-1 differs between sexes. Post-hoc analysis revealed that the acoustic parameter explaining 100% between-sex variability in Call-1 was the change in the frequency of the second part of the dominant frequency (Fig. 3B). Noticeable, the acoustic parameter enabling sex identification was different than the parameters coding individual variability of Call-1 (Fig. 3).

In DFA for sex we used only one call per individual to avoid pseudoreplication of the data and violation of the assumption of independence (explained in Methods section). To test the accuracy of classification to sex of all calls in our dataset (100 calls from 10 females and 140 calls from 14 males) we run a Logistic Regression, which showed 75.8% of correct classifications (74% of female calls and 77.1% of male calls). The outcomes of the regression using multiple calls per individual were similar to those obtained in DFA conducted on a single call per individual (75.8% vs. 79.2%), demonstrating notable sex differences of Call-1 irrespective of the method used in analysis.

**Figure 4:**
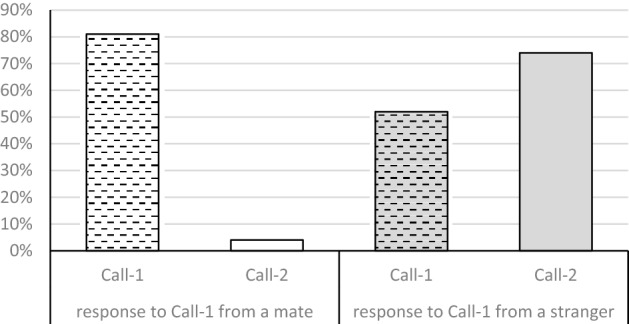
The response of paired individuals to the playback of Call-1 from their mate (white bars) and from a stranger (grey bars). The response of tested individuals with Call-1 is presented using hatched bars and with Call-2 using plain bars.

A play-back experiment in which 27 paired individuals of the Middle Spotted Woodpecker were presented either with Call-1 produced by their mate or Call-1 produced by a stranger revealed that territory owners responded differentially to both types of playback (Fig. 4).

A play-back of mate Call-1 in 81% of cases resulted in a vocal response with Call-1, which seems natural as Call-1 function as a contact call between pair members^12^. In only one case (which translates to 3.7%) a play-back of mate Call-1 was followed by an aggressive response with Call-2. On the other hand the play-back of stranger Call-1 triggered the response with both Call-1 (51.8%) and Call-2 (74%), and the latter one was more frequent. Bearing in mind that Call-2 warns the intruder about a territory owner’s readiness to a direct fight^12^, the results of the experiment indicate that territory owners discriminated between mate and stranger Call-1. The type of play-back (mate Call-1 vs. stranger Call-1) significantly affected the response with Call-1 (F = 4.07, *p* = 0.049) and Call-2 (F = 79.97, *p* < 0.001) of pair members.

## Discussion

Our study revealed that Call-1 of the Middle Spotted Woodpecker, which is the most frequent vocalization of pair members, is individually distinctive, exhibits significant sex differences and enables mate recognition. These results extend our previous findings demonstrating that Call-3, a characteristic vocalization of Middle Spotted Woodpecker floaters, is a vocal signature of unpaired individuals and enables their recognition by territorial neighbours^12^. Together these results show that, although the repertoire and the structure of Middle Spotted Woodpeckers calls is rather simple, it allows for coding identity and sex of a calling individual. Furthermore, the experimental approach revealed that woodpeckers make use of this information and distinguish between familiar individuals and strangers.

Although much has been written about vocal communication in birds^39^, the area of woodpecker *Piciformes* vocalization is still almost unexplored. The studies exploring vocal communication of woodpeckers not only are scarce but also they are descriptive^35,40^ and often involve observations of only few individuals^33,41^. Bioacoustic analyses and the experiment in our current study show that Call-1, which functions as a contact call between mates and a signal of a general arousal^12^, is individually distinctive and pair members respond differently when they hear a play-back of their mate Call-1 and stranger Call-1. In the first case they answer with Call-1, simply keeping in touch with the calling partner. This changes when it comes to a play-back of stranger Call-1, which mainly involves the response with aggressive and territorial Call-2 followed by Call-1. The presence of the latter one in the response towards a stranger may be explained by the fact that woodpeckers use Call-1 to express anxiety during any disturbance within a territory^12^ and also they might have called their mate to inspect the intruder. However, the response with Call-1 to the play-back of stranger Call-1 was significantly less frequent than a response with Call-1 to the play-back of mate Call-1.

Individual recognition based on vocal characteristics is widely described in to-date studies^16,42^. It was demonstrated not only in songbirds but also in species using less complex vocalisation^43–46^. A few studies described individual recognition by calls also in woodpeckers, including the Downy Woodpecker *Picoides pubescens*^47^ and the Accorn Woodpecker *Melanerpes formicivorus*^37^. Interestingly, woodpecker drumming was also demonstrated to be individually distinctive, however it was not tested whether it was used by birds for the recognition of conspecific^48^. Discrimination of individuals is beneficial because it enhances communication of mates over longer distances as well as it limits unnecessary, repetitive aggressive interactions between neighbours during the breeding season^49^. Thus one can expect that majority of vocalisation systems, including calls in woodpeckers, should provide the possibility of coding vocal signatures. We were not able to classify single calls to an individual with a 100% accuracy but our results are similar to those observed for call classification in other bird species, for example in African wood owls *Strix woodfordii*^50^, European nightjars *Caprimulgus europaeus*^46^, Corncrakes *Crex crex*^51^, or Woodcocks *Scolopax rusticola*^52^. It seems that call classification around 80% is a typical outcome in species possessing vocal signatures. Noteworthy, DFA in our study was conducted on single calls but during woodpecker vocalization Call-1 is often uttered in bouts, which likely rises the probability of individual identification of calling woodpeckers by their conspecifics to higher levels than indicated by DFA based on classification of single calls.

Our study also revealed that Call-1 uttered by males and females exhibits significant sex differences. Intersestingly, the acoustic parameter enabling sex identification was different than the parameters coding individual variability of Call-1, which shows that the structure of Call-1 is sufficient to transmit multidimensional information between individuals. The design of the call so that its first part would code the identity of an individual and the second part would code individual’s sex presents an effective and fine-tuned communication system. The results published by other researchers show that evolution of vocal communication in birds has led to a similar solution in a number of avian species. For example, the calls of the European eagle owl *Bubo bubo* and the Great horned owl *Bubo virginianus* encode information both on sex and ID of calling individual, however the acoustic parameters responsible for individual and sex differences were mostly the same and referred to frequency and temporal variables describing a call^53,54^. The study on Kittiwake *Rissa tridactyla* revealed that greeting calls produced by individuals are sexually dimorphic and individually distinctive and in this species both cues are included in different features of the call – sex of individual is encoded in the value of fundamental frequency of the call while ID in a set of temporal and frequency parameters of the call^55^. In many species of petrels, especially those active at night, calling is restricted to a single signal which conveys information about individual identity, sex and even a population to which the individual belongs^56^. In the Cory’s shearwater *Calonectris diomedea* female calls are characterized by lower fundamental frequency than male calls while ID of individuals is encoded in chronological ordination of syllables in a call^56^. In a play-back test birds discriminated both mates from strangers and males from females. Similarly, calls of the Manx shearwater *Puffinus puffinus* are sex and individually specific^57^. Sexual differences are encoded in the frequency spectrum, which is broader for females than for males. Individual differences include a set of temporal and frequency parameters of the call. Individual and sexual features were also demonstrated in begging calls of the Barn owl *Tyto alba* nestlings^58^. Interestingly, call features coding ID and sex became more pronounced with nestling hunger and increasing competition for food. In some species, like for example the White-faced whistling duck *Dendrocygna viduata,* differences between sexes were expressed significantly more strongly than the individual differences^59^. Also, the species were reported, in which individual features were absent in vocalization, however sexual identification of individuals was possible based on call parameters^60^. Above examples show that a number of solutions have been evolutionary designed to convey the information about an individual during vocalization. Our study adds to this variety by describing the system of coding ID and sex in the Middle Spotted Woodpecker. However, it should be underlined that further play-back experiments involving digital modification of woodpecker contact calls are needed to confirm our preliminary evidence that individual ID and sex are likely coded in separate acoustic parameters of a single call. Our study also lacks repeated recordings of the same individuals to confirm that vocal characteristics of woodpeckers encoded in a contact call are stable over a period of time. Thus further studies are needed to address above issues.

Taking into account that a waveform pattern of Call-1 is rather simple, one may expect that the number of combinations of features coding identity and sex is not endless, thus the efficiency for a proper ID coding may decrease with the increasing number of individuals tested. However, the Middle Spotted Woodpecker is rare, sedentary and does not live in high densities. Additionally, due to its habitat preferences towards old woods dominated by oaks it lives in small and isolated populations. It seems that in such systems Call-1 provides sufficient possibilities for proper individual recognition and identification. Interestingly, calls of a very similar structure to Call-1 of the Middle Spotted Woodpecker are present in vocal repertoires of other woodpecker species, for example the Lesser Spotted Woodpecker *Dryobates minor*, the Great Spotted Woodpecker *Dendrocopos major* and the Syrian Woodpecker *Dendrocopos syriacus* (Węgrzyn & Leniowski, *personal observ*.). It needs further investigation to test whether their function is similar, but if confirmed, it would reveal a simple and universal way of coding ID and sex across various woodpecker species.

The results of our study also suggest that Call-1 of the Middle Spotted Woodpecker, which is individually distinctive and shows notable sex differences, may be useful for conservation biologists as a tool supporting other census methods^61^. Sexing by voice could be conducted in the Middle Spotted Woodpecker only with some degree of probability because there is no acoustic parameter which does not overlap in ranges of values between sexes, however counting individuals based on spectral characteristics of their calls seems reliable.

## Conclusion

The contact call of the Middle Spotted Woodpecker is individually distinctive and woodpeckers are able to distinguish between a contact call of their partner and a stranger, suggesting that the call involves a vocal signature of paired individuals. The contact call also exhibits significant sex differences, although sexual features are less expressed than individual ones. The acoustic parameter enabling sex identification is different than the parameters coding individual variability of the call. Our study shows that the structure of the contact call is sufficient to transmit multidimensional information between individuals. The design of the call so that the first part will would code the identity of an individual and the second part would code its sex presents an effective and fine-tuned communication system. The results of our study also suggest that the contact call of paired Middle Spotted Woodpeckers may be useful for conservation biologists as a tool supporting other census methods.

## Material and methods

### The species

The Middle Spotted Woodpecker is a medium-sized woodpecker (20–22 cm long) of the Western Palaearctic that inhabits deciduous forests, especially areas with old oaks Quercus, hornbeams Carpinus and elms Ulmus, as well as a patchwork of clearings, pasture and dense woodland^62,63^. It is sedentary, socially monogamous^64^ and territorial in the spring^65^. Both parents share breeding duties^64^. Each year they excavate a new cavity and raise a single brood^63,66^. A relatively large red crown is characteristic of both males and females but is longer in males^38,62^ which enables sexing individuals by an experienced observer.

### Study area

The study was conducted in the riverine forest of Warta River Valley in Central Poland near Czeszewo (52°09′N, 17°31′E) in the years 2009–2010. The study plot consists of 222 ha of woodland with trees of the genera Quercus, Fraxinus, Ulmus, Fraxino and Ulmetum in flooded areas and Quercus, Carpinus, Stallario and Carpinetum on higher areas. The whole study area, of which about 40% is covered by mature, nearnatural forest stands, has been protected as the Czeszewski Las Nature Reserve since 2004.

### Territorry mapping and woodpecker status

Each year at the beginning of the breeding season (March/April) the entire study area was surveyed three times to search for breeding pairs and unpaired individuals^67^. To make surveys more efficient we used the play-back of Middle Spotted Woodpecker calls. All territories were mapped and confirmed during subsequent surveys. We distinguished between breeding and unpaired individuals based on an array of behavioral cues, such as: reaction to play-back (defence of a territory vs. retreat), presence/absence of a partner and a nest hole, presence/absence of persistent vocalisation typical for unpaired Middle Spotted Woodpeckers^63^. For more information on territory mapping and woodpecker status please refer to Węgrzyn & Leniowski^12^.

### Recordings and analyses of vocalization

Call-1 of Middle Spotted Woodpeckers was recorded using a shot-gun microphone AKG Blue line (SE 300 B + CK 98) with a windshield and a portable recorder Edirol R-09HR. Recordings were made in 2009–2010. We recorded Calls-1 during spontaneous vocalization of individuals. Call-1 is typically produced by individuals sitting on a branch or a trunk. In terms of social context it is used as a contact call with a mate without any obvious stimuli when pair-members forage separately in different parts of a territory, but it may also signal arousal due to any disturbance within a territory (e.g. a deer passing by or the flight of an aerial predator). Recordings were made between 9.30 a.m. and 2.00 p.m. as during the early spring this is the time of the highest vocal activity of Middle Spotted Woodpeckers (they become active with temperature rise). Calls produced by given individual were recorded on the same day but not in the same interaction. In analyses we used 10 calls per individual which were randomly selected from the calls recorded during our single visit to the territory.

### Acoustic analyses of Call-1 from different individuals

**Figure 5:**
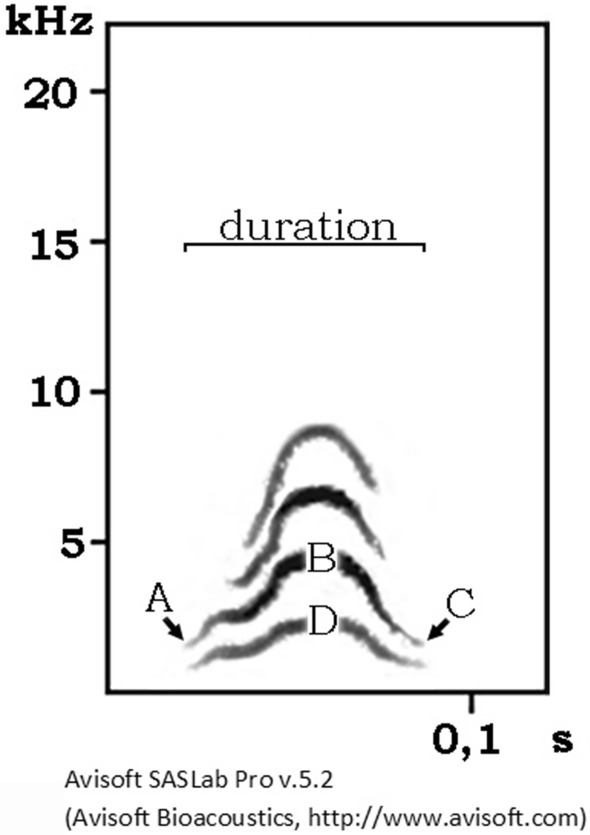
A sonogram of Call-1 and acoustic measures used to test whether Call-1 is individually distinctive. A: frequency of the beginning of the dominant frequency, B: frequency of the mid-part of the dominant frequency, C: frequency of the end of the dominant frequency, D: frequency of the mid-part of the fundamental frequency.

The visual analysis of sonograms of Call-1 revealed substantial individual differences in call parameters. Thus, we run thorough spectral analyses to investigate between- and within-individual variability of Call-1 using Avisoft-SASLab Pro software with the following parameters: 1024 FFT length, frame (%) = 25, window = Hamming and temporal overlap = 87.5%. This gave a 244 Hz bandwidth with 46 Hz frequency and 2.67 ms time resolution^68^. We used the following acoustic measures (Fig. 5): 1. duration of a Call-1, 2. frequency of the beginning of the dominant frequency (A), 3. frequency of the mid-part of the dominant frequency (B), 4. frequency of the end of the dominant frequency (C), 5. frequency of the mid-part of the fundamental frequency (D), 6. the change in the frequency of the first part of the dominant frequency (A/B), 7. the change in the frequency of the second part of the dominant frequency (C/B), 8. the change in the frequency of the whole dominant frequency (C/B–A/B).

**Figure 6:**
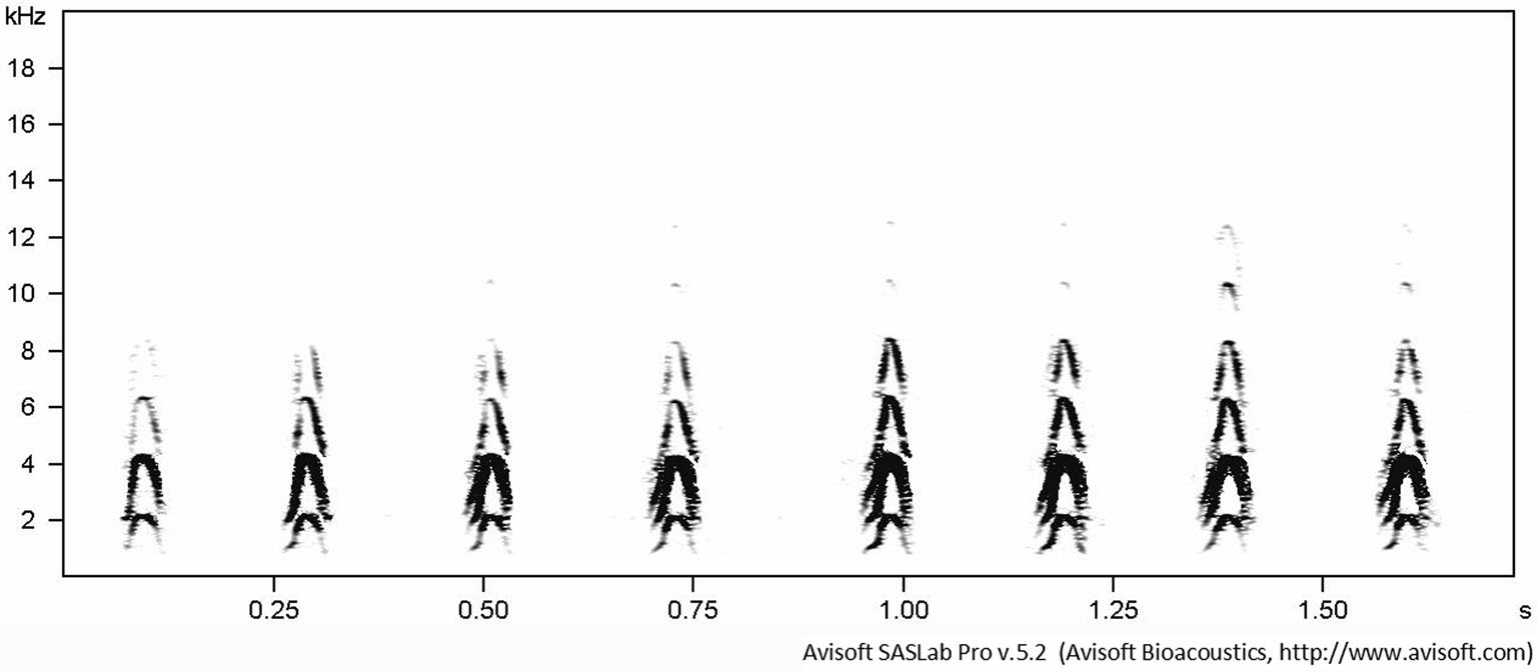
A sonogram of Call-1 uttered in a bout.

We measured 10 calls recorded from each of 31 paired individuals during our study. Call-1 may be uttered singly as presented in Fig. 5 but often it is repeated in a bout of variable length^12^ (Fig. 6).

The calls for measurements were extracted from the middle part of such bouts as the first and the last calls in each bout are often quieter and the measurement of their elements is less precise or sometimes impossible. In case of 24 recorded individuals we knew their sex (10 females and 14 males).

We used ANOVAs (with Bonferroni probability adjustments) to test if there were significant differences in the eight measured Call-1 parameters within and between individuals. We used a discriminant analysis (DFA) to determine whether Call-1 could be assigned to the correct individual based on the eight acoustic parameters described above. In the DFA we applied the cross validated classification and a leave-out-one test. DFA for ID was run on 310 calls from 31 individuals (10 calls per individual).

To test if Call-1 exhibits significant sex differences we compared the eight measured acoustic parameters of Call-1 produced by males and females using t-test. Subsequently we conducted DFA using the same parameters (with cross-validated classification) to determine the probability of the assignment of Call-1 to the correct sex. However, DFA for sex was run on a single call per individual (10 female calls and 14 male calls, each call was randomly chosen from a pool of 10 calls produced by given individual) because using multiple calls from a given individual when trying to classify the calls into some category other than individual ID (such as sex) can bring misleading results. If one tries to use traditional DFA in this scenario, the assumption of independence will be violated and the results may be misleading. Doing so would pseudoreplicate the data and hence violate the assumption of independence^69^. In the case of purely nested data comprising only two factors (like ID and sex) the solution is to use only a single randomly selected call per each subject or to use a Logistic Regression which is less restrictive than DFA with regard to assumptions, permits the analysis of multi-factor data sets, and also allows for a classification of cases^70^. However, Logistic Regression does not allow for a stepwise method which reveals the parameters that explain the highest level of between-class variability. As we wanted to know which call parameters differ the most between the sexes and at the same time we also wanted to test the accuracy of classification to sex of all calls in our dataset we run both analyses (i.e. DFA with the stepwise method using a single call from each male and female and Logistic Regression using 10 calls from each of 10 females and 14 males).

### Experiment

The aim of the experiment was to test the response of paired individuals to Call-1 produced by their mates and by strangers. The experiment was conducted in the year 2010 before egg laying stage (March/April) when the vocal activity of the Middle Spotted Woodpeckers is the highest. We conducted play-backs of Call-1 of a pair-member and a stranger in 27 breeding territories occupied by Middle Spotted Woodpecker pairs. Stranger play-backs were prepared using vocalization of individuals that occupied distant territories from the subjects of the experiment. The study plot consisted of 222 ha of woodland and Middle Spotted Woodpeckers are sedentary, so an individual from a distant territory (whose vocalization was used to prepare the play-back) was considered a stranger for the territory owner to whom the play-back was presented. Responses to play-backs were recorded, transformed to sonograms using Avisoft-SASLab Pro software and analyzed. To prepare play-backs we used recordings of Call-1 collected earlier in the same population. The playbacks of mate Call-1 and stranger Call-1 presented in the same location were separated by an interval of at least 72 h. The play-back of mate Call-1 and stranger Call-1 were presented in alternating order in subsequent locations. To avoid pseudoreplication, each woodpecker received a play-back of Call-1 of a stranger prepared from the recording of a different individual. Playbacks of Call-1 consisted of five bouts, 15 calls in each bout (to mimic a spontaneous production of these calls described in Węgrzyn and Leniowski^12^ as close as possible). Bouts of calls used in the play-back experiment were natural recordings of Call-1 adjusted to the length of 15 calls per bout by deleting final calls if an original bout exceeded 15 calls. We did not elongate too short bouts by inserting additional calls as we wanted to minimize manipulation. Bouts were separated by 5 s of silence (mean inter-bout interval in Call-1 from a natural vocalization of the Middle Spotted Woodpecker was 4,8 s ± 2.48). We put in effort to do play-backs in such a way as to avoid the subject’s mate presence during the experiment. It was possible because in the studied population of the Middle Spotted Woodpecker each mate forages in a different part of the territory of the pair^69^. We did play-backs within a subject’s foraging range and the distance between the speaker and the tested woodpecker was short (30–40 m), so the subject bird was always the first who responded to the play-back. In all cases we were able to see the subject before and during the play-back. The play-back was turned off as soon as a subject individual started responding to avoid masking the response by the play-back. To test whether the play-back of mate Call-1 and stranger Call-1 triggers a different response from territory owners we conducted a Linear Mixed Model with REML method and main effects design. We used repeated measures approach with the type of a playback as a fixed factor, the sex of an individual as a random factor and the type of a response (Call-1 and Call-2) as dependent variables. A repeated measure procedure was applied because each woodpecker was tested twice (with a play-back of mate Call-1 and stranger Call-1).
